# Clinical Reasoning Curricula in Health Professions Education: A Scoping Review

**DOI:** 10.1177/23821205231209093

**Published:** 2023-10-25

**Authors:** Maria Elvén, Elisabet Welin, Desiree Wiegleb Edström, Tadej Petreski, Magdalena Szopa, Steven J. Durning, Samuel Edelbring

**Affiliations:** 1 School of Health, Care and Social Welfare, 8177 Mälardalen University, Västerås, Sweden; 2 Faculty of Medicine and Health, School of Health Sciences, 6233 Örebro University, Örebro, Sweden; 3 Faculty of Medicine and Health, School of Medical Sciences, 6233 Örebro University, Örebro, Sweden; 4 Institute for Biomedical Sciences, Faculty of Medicine, 54765 University of Maribor, Maribor, Slovenia; 5 Department of Nephrology, Clinic for Internal Medicine, 112806 University Medical Centre Maribor, Maribor, Slovenia; 6 Department of Metabolic Diseases, 49573 Jagiellonian University Medical College, Krakow, Poland; 7 Department of Medicine, Center for Health Professions Education, 1685 Uniformed Services University of the Health Sciences, Bethesda, MD, USA

**Keywords:** clinical reasoning, clinical decision-making, curriculum design, health professions education

## Abstract

**OBJECTIVES:**

This scoping review aimed to explore and synthesize current literature to advance the understanding of how to design clinical reasoning (CR) curricula for students in health professions education.

**METHODS:**

Arksey and O’Malley's 6-stage framework was applied. Peer-reviewed articles were searched in PubMed, Web of Science, CINAHL, and manual searches, resulting in the identification of 2932 studies.

**RESULTS:**

Twenty-six articles were included on CR in medical, nursing, physical therapy, occupational therapy, midwifery, dentistry, and speech language therapy education. The results describe: features of CR curriculum design; CR theories, models, and frameworks that inform curricula; and teaching content, methods, and assessments that inform CR curricula.

**CONCLUSIONS:**

Several CR theories, teaching, and assessment methods are integrated into CR curricula, reflecting the multidimensionality of CR among professions. Specific CR elements are addressed in several curricula; however, no all-encompassing CR curriculum design has been identified. These findings offer useful insights for educators into how CR can be taught and assessed, but they also suggest the need for further guidance on educational strategies and assessments while learners progress through an educational program.

## Introduction

To do the right thing in the right way at the right time in clinical contexts, health professionals need clinical reasoning (CR) abilities. CR is thus an essential component of health professional's practice and includes knowledge and skills to gather and analyze patient data, arrive at diagnoses, and make management decisions in relation to patient needs, often under time pressure. A broad definition of CR is “a context-dependent way of thinking and decision-making in professional practice to guide practice actions.”^1^ This implies conscious and nonconscious cognitive and metacognitive activities that interact with patients’ unique situations and preferences and with contextual factors such as practice environments.^2^ In clinical practice the health care provider performs CR by, for example, taking a patient history, performing a physical examination and ordering lab tests, interpreting these data to arrive to a diagnosis, and deciding on medical treatments and other management approaches. This CR process includes an interplay between the health care provider(s), the patient, and next of kin.^3^ Different professions engage to various degrees in these processes prior to and following reaching a diagnosis. Health professions curricula have supported training in CR under additional labels such as diagnostic skills, caring decisions, and rehabilitation strategies. However, few strategies for outspoken CR teaching have been described. The literature shows instead that CR is scarcely explicitly taught or assessed in health professions education programs.^4–8^ Reasons for this scarcity could be CR's complexity, multidimensionality, and varying definitions within and across professions.^9^ As CR in practice is not a straightforward single best route to arrive to a diagnosis or a management decision, CR teaching and learning implies consideration of ambiguity and a focus on the process, not only the outcome, which might be challenging to apply in nonclinical learning contexts.^10^ Barriers to more specifically addressed CR teaching include a lack of awareness of teaching and assessment methods and a change-resistant teaching culture, implying curricula dominated by memorizing factual knowledge and focusing on correct diagnoses rather than training the reasoning process.^11^ Without explicitly targeting CR in teaching, CR risks to become a “black box” phenomenon^12^ that students need to explore by themselves.^6^

Given CR's core nature and relation to patient safety and the ability to meet multiple care needs,^13^ structured CR curricula should be incorporated into all health professions educational programs. A survey conducted in 76 countries revealed that such a curriculum was only reported by a third of the educators, while most expressed the need for implementing such an explicit CR curriculum.^5^ The term curriculum concerns what is taught, how it is taught, when it is taught and how the students’ learning are assessed.^14^ Curriculum planning frameworks emphasize the importance of interrelating or unifying subjects frequently taught in separate courses or modules to make a meaningful whole.^15,16^ Thus, to teach CR explicitly and systematically, it should have a clear curricular strategy integrated throughout the education curriculum.

Incorporation of such a CR curriculum is needed across all health professions education, but little guidance exists. A UK consensus group^17^ suggested the application of 5 “whats” and related teaching strategies in medical education. A European collaboration^18^ taking a multiprofessional approach also presented a framework proposing educational strategies to frame a CR curriculum. Some initiatives have described local and profession-specific CR curricular strategies.^19–21^ How to structure CR curricula throughout educational programs is thus a complex challenge and further guidance is needed. However, no body of literature has reviewed current designs of CR curricula in health professions education specifically and thus the present review aims to fill that gap.

The aim of this scoping review is to explore and synthesize current literature to advance the understanding of how to design CR curricula for students in health professions education. Three review questions guide the scoping review: (I) What are the features of CR curricular design in health professions education? (II) Which theories, models, or frameworks of CR are used in current CR curricula, and how have these informed curricular design? (III) What teaching content, methods, and assessment forms exist in CR curricula, and how have these informed CR curricular design?

## Methods

### Design

A scoping review was selected because it is recommended for complex fields of interest that have not been comprehensively reviewed.^22^ The systematic 6-stage methodological framework for a scoping review by Arksey and O’Malley,^22^ and further enhanced by Levac et al,^23^ was used. A study protocol has been previously published.^24^

### Identifying relevant studies

*Eligibility criteria.* Peer-reviewed journal articles including original qualitative, quantitative, and mixed-methods research, reviews, commentaries, and guidelines were included. The articles should be published in a recent 10 years span [2010-2020] to cover contemporary views on CR in health professions education as the number of articles on clinical reasoning across health professions literature has grown extensively during the last decade.^9^ Eligible articles were in English, describing a CR curriculum framework or its aspects, and/or describing and setting important CR aspects (eg, gathering information, generating a diagnose, diagnostic errors, treatment planning, and collaboration with the patient and team), methods, or examples into a curricular perspective, and/or contributing knowledge and/or examples with value for developing a CR curriculum framework. The CR curriculum frameworks, aspects, teaching methods, or examples needed to be connected to a full educational program or address at least 2 semesters, courses, or modules of a program. Articles from all health professions educations and countries were eligible. Articles were excluded if they dealt with CR aspects, models, teaching methods, and/or specific examples (eg, specific organs or diseases) without a connection to an explicit curriculum. Furthermore, articles were excluded if they described CR in relation to a single course or module of an educational program, for example, one specific semester of an educational program or a 10-week course/module focused on a specific topic.

*Information sources and search strategy.* The adapted search strings, presented in Appendix 1, were applied in PubMed, Web of Science, and CINAHL, initially on April 28, 2020, and updated on October 10, 2020. In addition to the search strategy, manual searches in, for example, Google Scholar and snowballing through reviewing the reference lists of included studies were performed.

### Study selection

One author (SE) conducted the searches and imported the references into the Covidence systematic review software (Veritas Health Innovation, Melbourne, Australia). The initial title and abstract screening was performed by a larger team involved in CR educational work and research. A consensus decision for eligibility was performed by a combination of 2 team members. In case of uncertainty or disagreement, one of the core teams (ME, EW, and SE) made the decision. Each full-text article was thereafter assessed for inclusion by 2 authors (any combination of ME, EW, SD, and SE). Uncertainty or disagreement were solved in consensus discussion and using a third author as arbiter when needed. A second assessment round of the included full-text papers was conducted to ensure precision. Because of the review's scoping nature, focusing on relevant content and not the study design or quality of findings, no quality appraisal was performed.^25^ The PRISMA flowchart^26^ was used to report the study selection process.

### Charting the data

A data charting form for data extraction was developed, piloted on 5 articles, and refined by 3 of the authors. The included articles were then distributed among the authors, who extracted data independently using the form. In case of uncertainty, any of ME, EW, or SE reviewed the data and a consensus decision was made. Final consensus of the data, presented in tables and text, was reached with all authors. If an author's own article was analyzed, others on the reviewing team performed data extraction and primary analysis.

### Data analysis

Characteristics of the studies including author(s), publication date, country, aim(s), study design, health profession, and concept(s) were descriptively presented. Extracted data were analyzed and grouped based on the 3 review questions in a primary analysis performed by 3 authors (ME, EW, and SE) and then discussed and refined collaboratively with all authors. In addition, structures established in work on needs analysis of CR curricula^5^ and assessment of CR^27^ guided categorization of data concerning teaching content, methods, and assessment forms that inform CR curricula. The PRISMA-ScR checklist^28^ was used for the reporting of results.

### Consultation

The preliminary findings were shared with 2 external stakeholders, who are medical and nursing educators with extensive experience in CR and curriculum development in 2 different European countries. They were asked to review the results and discussion and provide feedback on topics that should be highlighted and the findings’ main implications.

## Results

### Study selection

**Figure 1.**
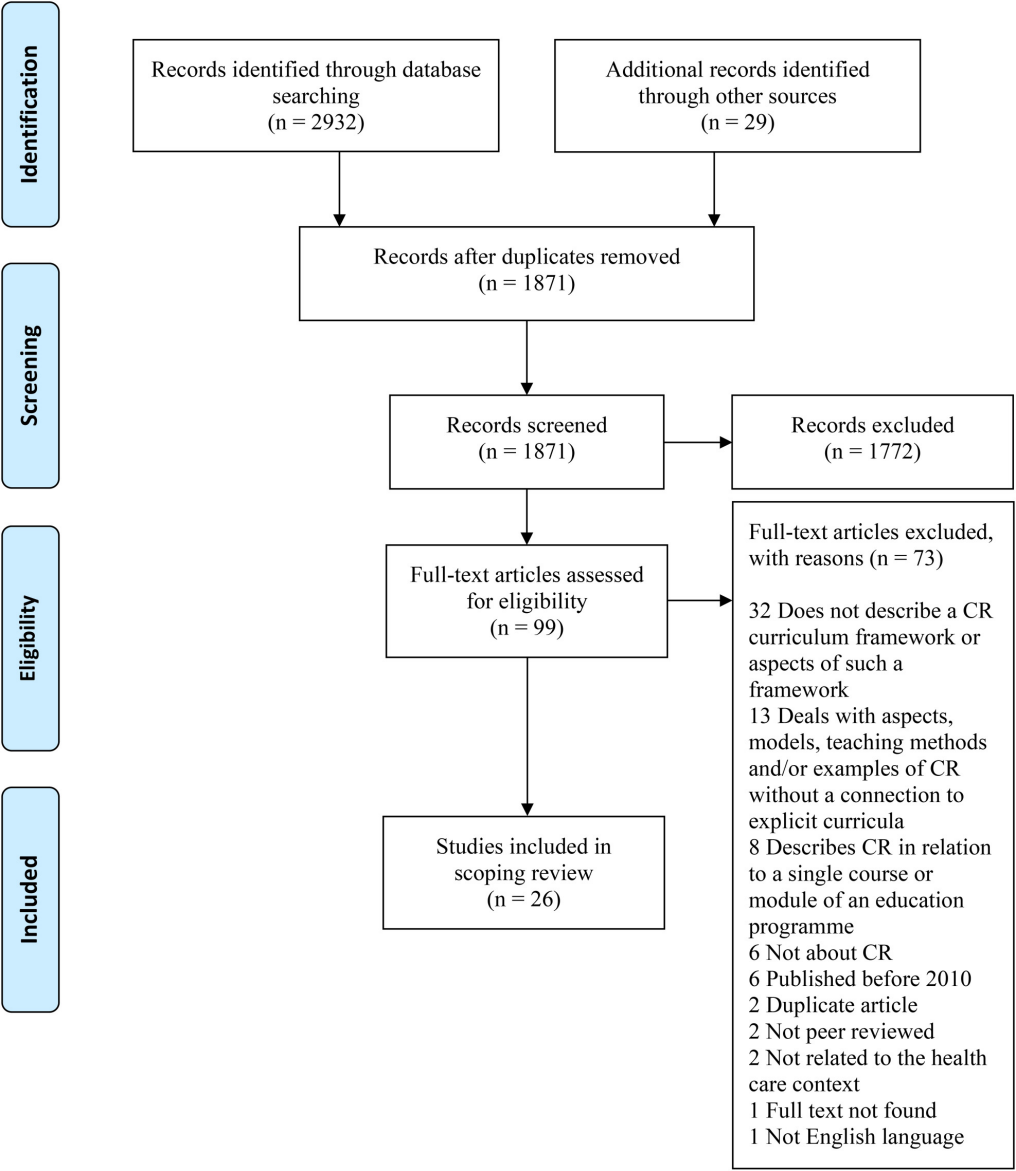
PRISMA flow diagram.

The search revealed 2932 studies and 29 studies were added manually. After screening and full-text assessment, 26 articles were included. Figure 1 shows the process in a PRISMA diagram.

### Study characteristics

The included studies described aspects of CR and their integration into health professions education curricula. Appendix 2 presents an overview. The articles included medical, nursing, physical therapy, occupational therapy, midwifery, dentistry, and speech-language therapy education.

### Features of clinical reasoning curriculum design

No articles presented the development or implementation of a CR curriculum design covering a full educational program. A range of theories, models, and learning activities was described in relation to parts of curricula. Some studies stressed the importance of integrating CR teaching and assessment across the curriculum.^5,6,20,21,29,30^ Dowding et al^19^ argued that CR curriculum design should adapt to students’ progression from being a novice to being competent, to the organizational context, such as guidelines, development rules, and regulations, and to the actors in the reasoning process, such as patients, relatives, and team members. Several studies, for example,^19,30–32^ emphasized that teaching focus on developing and facilitating sound decision-making process skills in all CR phases: data gathering and interpretation, diagnostics, and treatment planning. Various teaching models and learning activities were proposed for systematic use. Posel et al^33^ and Khin-Htun and Kushairi^34^ presented activities aimed at enhancing students’ CR development throughout a program, including ward-based learning, case methodology, symptom-focused teaching, and virtual patients. An overall approach to structure teachers’ support for students while engaging in realistic clinical scenarios in all curriculum stages was proposed by Hensel and Billings.^30^ Case methodology,^35^ problem-based learning (PBL),^19^ and graphical representation of CR using knowledge-modeling software^36^ were recommended to support overarching CR educational principles. To stimulate development of CR abilities, Pinnock and Welch^37^ emphasized targeting unaware aspects of CR throughout the curriculum, and Furze et al^20^ similarly emphasized students’ self-reflection.

### Clinical reasoning theories, models, and frameworks that inform curricula

Several CR theories and models for CR development were presented. Dual-process theory^6,29,37–40^ and script theory^6,29,36,41,42^ were frequently cited for curricular development and to explain CR processes. Tanner's model and related lines of thought in Benner's model of clinical judgment also appeared.^30,31,43^ Less mentioned were hypothetico-deductive reasoning, situativity theory, deliberate practice theory and metacognition, the CR cycle by Levett-Jones,^44^ the CR model focused on clients’ behavior change with reference to physiotherapists (CRBC-PT) by Elvén et al,^45^ the Outcome-Present State Test (OPT) model by Kuiper,^21^ Brooks’ theory of intrapersonal awareness, and cognitive load theory. Some models connected learning activity design. Furze et al^20^ used the Dreyfus model for skill acquisition for mapping CR phases, relating these to consequences for CR curriculum design and progression. Schmidt and Mamede^40^ categorized teaching approaches into 2 major theoretical strands to teach CR using patient cases, serial cues, and the whole cases. Other examples are the Patient/Client Management Model, the Hypothesis-Oriented Algorithm for Clinicians, and the International Classification of Functioning, Disability, and Health model.^46^

Frameworks and models to support CR characteristics were also described. Nafea and Dennick^41^ used a modified SPICES framework (student-centered, problem-based, integrated, community-based, elective, and systematic approach) and integration ladder^15,16^ to contrast different curricular integration of CR, using comparative items (eg, student-centered vs teacher-centered and elective vs uniform) to evaluate CR's curricular presence. They found curricular differences in, for example, student centeredness, the start of clinical exposure, and its degrees. Kantar and Alexander^31^ used Tanner's concepts of noticing and interpreting to assess nursing curricula, and Stickley^46^ used the Fink model to develop a CR curriculum. Marcum^38^ suggested the dual-process model, to increase curricular awareness of CR. As Menezes et al^47^ and Kononowicz et al^5^ conclude, consistent CR teaching throughout curricula is also important, and faculty development and competence in CR is central.

### Teaching content, methods, and assessment forms that inform clinical reasoning curricula

Table 1 presents the teaching content, (ie, what teaching in CR entails), and Table 2 shows the teaching methods (ie, how CR is taught). The teaching content focused on gathering, interpreting, and synthesizing information and generating a diagnosis and/or differential diagnoses, while CR's interprofessional aspects were least described. Case-based learning and workplace-based learning dominated. Simulation, including scenarios where students tackle increasingly complex care and decision-making,^19^ virtual patients, (ie, a computer-based simulations of a clinical scenarios^33^), and flipped classrooms,^32^ were also common. Dowding et al^19^ presented the benefits of the PBL structure and emphasized the necessity of academic-clinical partnerships.

**Table 1.** Clinical Reasoning Teaching Content (What Teaching in Clinical Reasoning Entails).

REFERENCE	GATHERING, INTERPRETING AND SYNTHESIZING INFORMATION	GENERATING A DIAGNOSE AND/OR DIFFERENTIAL DIAGNOSES	DEVELOPING A TREATMENT PLAN	SELF-REFLECTION ON CLINICAL REASONING PERFORMANCE	ERRORS IN THE CLINICAL REASONING PROCESS	BIASES IN CLINICAL REASONING	ASPECTS OF PATIENT PARTICIPATION/PATIENT-CENTERED APPROACH	INTERPROFESSIONAL ASPECTS OF CLINICAL REASONING	THEORIES OF CLINICAL REASONING	OTHER
Charlin et al^36^	X	X	X	X						
Christensen et al^48^	X	X	X	X			X			
Dalton et al^32^	X	X	X	X			X			
Daniel et al^27^	X	X								
Dowding et al^19^				X	X	X	X	X	X	a)
Elvén et al^49^	X	X	X	X			X			b)
Findyartini et al^42^		X				X				c)
Furze et al^20^				X						
Gillespie^43^	X	X	X			X	X			d)
Harendza et al^4^	X	X		X	X	X				
Hensel and Billings^30^	X	X	X	X	X	X	X			
Kantar and Alexander^31^	X	X	X	X						
Khin-Htun and Kushairi^34^	X	X	X				X			
Kononowicz et al^5^	X	X	X		X			X	X	e)
Kuiper^21^	X	X	X	X	X	X	X	X	X	f)
Marcum^38^	X	X		X					X	
Menezes et al^47^				X					X	
Nafea and Dennick^41^				X			X			c)
Orban et al^35^	X	X	X					X		
Pinnock and Welch^37^	X	X			X	X				
Pinnock et al^39^	X	X			X	X				
Posel et al^33^	X	X	X	X	X		X			
Rencic et al^6^	X	X	X	X	X	X			X	
Schaye et al^29^	X	X		X		X			X	
Schmidt and Mamede^40^	X	X								
Stickley^46^	X	X	X	X	X	X	X			

(a) The importance of feedback; the nature and role of expertise; decisions aids; system 1 and system 2 in thinking; Pattern recognition; Scenario-based thinking; perception and communication of risk. (b) Curricula with behavioral medicine competencies integrated in clinical reasoning. (c) Influences of culture on clinical reasoning teaching and learning. (d) Meta-cognitive reflection in “knowing the self”. (e) Strategies to learn clinical reasoning. (f) Reflective journaling.

**Table 2.** Clinical Reasoning Teaching Methods (How Clinical Reasoning Is Taught).

REFERENCE	PROBLEM-BASED LEARNING	SIMULATION	CASE-BASED LEARNING	VIRTUAL PATIENTS	WORKPLACE-BASED LEARNING OR BED-SIDE TEACHING	LECTURES	ROLE MODELING	DIGITAL TOOLS, EG, VIDEOS	OTHER
Charlin et al^36^									
Christensen et al^48^					X	X			
Dalton et al^32^		X				X		X	a)
Daniel et al^27^									
Dowding et al^19^	X	X	X						b)
Elvén et al^49^									
Findyartini et al^42^	X								c)
Furze et al^20^									
Gillespie^43^			X		X				
Harendza et al^4^			X			X			
Hensel and Billings^30^			X		X				d)
Kantar and Alexander^31^		X	X			X			
Khin-Htun and Kushairi^34^	X	X	X	X	X	X	X		
Kononowicz et al^5^	X			X	X	X			
Kuiper^21^			X		X				e)
Marcum^38^					X		X		f)
Menezes et al^47^		X	X		X			X	g)
Nafea and Dennick^41^	X								g)
Orban et al^35^			X						
Pinnock and Welch^37^			X	X	X				h)
Pinnock et al^39^									
Posel et al^33^				X					
Rencic et al^6^			X	X	X			X	
Schaye et al^29^			X		X				
Schmidt and Mamede^40^			X						i)
Stickley^46^			X		X				

(a) On-line lectures, flipped classroom (b) Timely feedback; adaptation to the development process from novice to advanced beginner to competent (c) consideration of power distance (d) prompts (e) OPT model worksheet (f) need a coach/mentor and longitudinally observe to be consistent with deliberate practice (g) promoting student reflection (h) Case reports and think aloud techniques (i) The serial-cue approach; the whole-case approach: knowledge-oriented approach.

The assessment methods were diverse. Work-based assessments included clinical instructors’ ratings of students’ clinical skills performance,^21,46,48^ mini clinical evaluation exercises,^5,37^ and the Physical Therapist Clinical Performance Instrument.^48^ Nonwork-based assessments included students’ self-reported confidence in making clinical decisions in practical exams^46^; self-reflections^21^; self-reported CR skills^4^; written assessments, such as the Script Concordance test,^20,37^ the Key Feature approach,^5^ the Diagnostic Thinking Inventory,^29,42^ the Reasoning 4 Change instrument,^49^ critical thinking tests,^20,47^ and the National Council Licensure Examination for nurses^30^; written case reports,^4,29,37^ think-aloud techniques,^20,37,41,43^ and observational tools used together with the case method.^35^ Assessment in simulated environments included the use of virtual patients in which feedback, and self- and formative assessments were integrated,^33,37^ and objective structured clinical examinations.^5^ Moreover, Daniel et al^27^ provided numerous examples of work-based, nonwork-based assessments, and assessments in simulated environments that can be applied.

How teaching content, teaching methods, and assessment forms were integrated and used in the design of CR curricula varied according to, for example, time in the educational program and context. Awareness of potential cognitive errors, decision biases, and questioning one's own actions and decisions in light of this knowledge was a recommended teaching approach in CR curricula designs.^6,19^ Harendza et al^4^ emphasized that cognitive errors need to be made explicit to students to reveal how such errors can influence the diagnostic process. Recommended methods to teach about errors and biases are that clinicians slow down and think aloud as they progress through a case and that students explain how they are thinking about their reasoning process.^39^

Patient participation in the CR process was emphasized in addition to teaching content, for example,^19,21,46^ and practicing CR abilities was recommended to be performed in patient encounters.^21,34^ How patients’ contexts, needs, and preferences could influence a problem's presentation, examination, analysis, and management plan was emphasized.^46,49^ Dowding et al^19^ emphasized that students need to understand team influences in CR and errors and biases that can be inherent. According to Orban et al,^35^ students, especially in their later years of the education, have the prerequisites to include other professionals’ perspectives in their CR.

Active learning strategies and experiential training to engage students in CR training components were stressed as key in curriculum design.^31,46^ Such training can include case-based learning, small-group discussions,^6,31^ flipped classrooms,^32^ simulations,^31^ and digital support, such as virtual patients.^33^ The incorporation of artificial intelligence into preclinical courses was highlighted as a future possibility to facilitate learning of, for example, information gathering, diagnostics, and patient-centeredness.^39^ Findyartini et al^42^ demonstrated the need to address cultural issues for effective CR teaching and learning. Thinking aloud, questioning, or using prompts to elicit cognitive behaviors were recommended to support self-reflection. Batteries of prompts and questions to be used in the decision-making process were presented by the National Council of State Boards of Nursing^30^ and in the situated clinical reasoning framework.^43^ Ample feedback was also highlighted to help students grow in their CR skills throughout the curriculum.^21^

Christensen et al^48^ demonstrated that common CR assessment methods in American physical therapy education programs lacked specificity for various CR aspects and were also often specific to single programs. Daniel^27^ emphasized the incorporation of various assessments targeting different components of the CR construct into curricula and recommended assessment programs. Few studies presented assessments adapted to the complexity of CR and students’ CR development. The situated clinical decision-making framework presented by Gillespie^43^ was suggested as a basis for the examination of different phases of nurse students’ clinical decision-making processes and as an exploration of their CR in a wider context. By following the framework's structure, key considerations related to foundational knowledge, thinking, the reasoning process, and the context could be implemented in CR assessments throughout the curriculum. Furze et al^20^ developed a conceptual CR framework on the basis that physical therapy students develop CR gradually. Early in their education, students are internally focused and are challenged in integrating new information. Later, external awareness evolves, and by the end of their education, students have developed reflection skills. The authors concluded that these phases need to be considered in assessments. Orban et al^35^ highlighted the case method as suitable for monitoring students’ CR progression and evaluating the curriculum quality. According to Kononowicz et al,^5^ there is a gap in the use of work-based assessments, (ie, real-life patient scenarios), while nonwork-based assessments and simulated situations are adequately represented. Moreover, the workplace context is emphasized as a relevant arena in which to assess CR.^27^

## Discussion

This review aimed to explore and synthesize current literature to advance the understanding of how to design CR curricula in health professions education. Our review is the first to address CR curricula across health professions. The results describe features of CR curriculum design; theories, models, or frameworks of CR that inform curricula; and teaching content, methods, and assessment forms that inform CR curricula. The findings highlighted that no longitudinal model for CR progression ready for implementation was presented, and concrete guidelines for designing a curriculum supporting progression of CR are lacking. Some studies portrayed how specific CR elements should be targeted in CR curricula, including features related to students’ development of knowledge and experiences in CR across time, as described by Dowding et al^19^ and Furze et al^20^ Since students early in their education focus on their own performance and, to a large extent, are limited to isolated components within their CR, they need rules to guide their CR practice. Later, students can widen their perspective to include more components in their CR, identify patterns in clinical situations, and subsequently deal with more complex cases that rely on their analytical thinking and reflection. Use of a “serial-cue” approach (ie, cases are unfolded to students gradually), at the beginning of education followed by a “whole-case” approach is one way to meet differences in students’ learning development.^40^ Another element was contextual features, including organizations, patients, and others involved in care and treatment. Dowding^19^ emphasized that curriculum design needs to address CR's occurrence in a context by targeting the importance of patient needs and the influences of teams and regulations. This implies that CR curricula need to ensure that health professional students learn how to react to a situation and adapt their reasoning accordingly. A further element was related to phases or dimensions in the CR process that should frame CR curricula. Different models or labels were used to describe the CR process. For example, Kantar and Alexander^31^ highlighted the dimensions of judgment by Tanner^50^: noticing, interpreting, responding, and reflecting. Dalton et al^32^ described the CR cycle by Levett-Jones^44^: collecting cues, processing information, identifying problems, establishing goals, taking action, evaluating outcomes, and reflecting. CR abilities should evidently form the basis of curricula; however, guidance on teaching different CR phases over time is scarce.

Our analysis conveyed the impression that learning activities and their outcomes dominated the studies’ data rather than the CR theories and models that underpin the activities. However, models to support curricular development included SPICES and the Fink model. Although not targeting CR per se, such models help educational designers consider the larger picture and support CR progression. Efforts such as the European DID-ACT collaborative^5^ and the Manchester Medical School program^51^ provide examples of designing CR educational support from a longitudinal perspective. Our sample represented studies referring to models and theories explaining CR and CR education along with empirical data on their implementation. One approach does not fit all, and different approaches may be effective in different stages of CR progression. Dual-process theory, the most prevalent theory in our study, acknowledges 2 important aspects of CR: experiential pattern recognition and a deliberate analytic process. Eva^10^ suggested designing activities with both approaches (ie, to support students’ development of underlying factual knowledge and clinical experiences that support pattern recognition). The findings of this review reveal a mix of CR teaching methods including experiential training such as case-based and workplace-based learning, and more passive learning such as lecturing. Active learning methods prevailed. Mixing the methods could imply a deliberate strategy to support both pattern-recognition and analytic approaches to CR. As CR is differently conceptualized across health professions, educators need to make the intended meaning of CR explicit in their curriculum work.^9^ Different traditions and CR models seem to be productive in respective contexts, such as the OPT model in nursing and the International Classification of Functioning, Disability and Health, and CRBC-PT model in physical therapy. However, to meet the future needs of team collaboration in increasingly more prevalent complex clinical scenarios, CR models addressing interprofessional collaboration are needed. The CR interprofessional competency framework by Olson et al^13^ is one example of supporting health professions education programs to improve CR teamwork.

The data revealed many teaching and assessment methods for CR activities, providing educators with the possibility to choose methods according to their context based on informed decisions. Conversely, the variety of methods can result in uncertainty regarding prioritization of what, how, and when CR should be taught and assessed. Given that many educators perceive a lack of CR competence, faculty development is needed.^5^ Active learning strategies and experiential training were identified as vital to support students’ CR skills based on students’ and teachers’ experiences and the collective view of scholars. Evaluation of student CR outcomes demonstrated the effectiveness of active learning for students’ confidence in clinical decision-making^46^ and progress in CR abilities.^4^ Active learning focuses on observing, doing, and reflecting on what was learned and how it was learned, as opposed to more passive forms of learning such as listening to a lecture.^46,47^ This finding is consistent with the consensus-based recommendations for CR teaching in medical education.^17^ Higher education research demonstrates that students are more receptive when they engage with their learning.^52,53^ However, active learning requires increased cognitive efforts, and students can feel like they learn less compared to a more passive approach.^54^ As CR predominately relies on advanced cognitive activities,^36,38^ it is paramount that students appreciate the benefits of putting effort into their active learning and not lose motivation for learning. Most of the included methods, such as case-based, ward-based, and problem-based learning, and think-aloud in collaboration with peers or clinical supervisors, correspond well with attributes that support perceived meaningfulness in learning.^55^ However, many curricula are still lecture-based.^5^ Lectures should not necessarily be removed but used to link theory with CR practice, specifically in early education,^34^ and be complemented by active methods.

CR teaching content in health professions education is dominated by the CR process, starting with examination and ending with a treatment plan, which is in agreement with the consensus of medical educators.^17^ Our findings also emphasize self-reflection. Reflection on one's own cognitive processes and performance is vital, as metacognition is integrated into CR's nonanalytical and analytical processes.^38^ Most studies implicitly assumed that CR occurs with a focus on the individual professional in relation to the clinical situation. Few studies provided examples of teaching content in which patients and relatives were empowered to participate in the CR. This could be explained by a dominance of CR's diagnostic aspects with a right answer based on biomedical-oriented symptoms.^56^ Further guidance is needed to help educators teach about interacting situation-specific factors, such as patients’ characteristics, needs, and preferences and the clinical context, that are essential in CR.

The importance of context in CR, as opposed to cognitive processes only,^1^ was emphasized in CR assessments. Educators are encouraged to focus less on a single best route when assessing students’ reasoning process and more on the impact of context and multiple possible answers.^2^ Increased use of work-based assessments and simulations is recommended.^27^ Singh et al^51^ proposed Miller's Prism of Clinical Competence^57^ to direct assessments from factual knowledge toward actions. Similarly, Daniel et al^27^ recommended the implementation of an assessment program to cover different components of the complex CR construct throughout curricula. Recommendations about which assessment methods address different stages in students’ CR learning progress best are warranted.

### Strengths and limitations

A strength is the inclusion of articles from different health professions CR curricula, thereby initiating a coherent view of CR teaching strategies. The interdisciplinary nature of the study provides educators with many examples of CR teaching and assessment that could be directly, or with minor adjustments, applied in future curricula design. The research team included researchers from diverse backgrounds (clinicians and educators from 4 countries) and multiple professions (medicine, nursing, and physical therapy), implying that different perspectives were considered, which is considered a strength for this review's multiprofessional focus. The inclusion of external stakeholders provided important feedback on the findings, which improved the usefulness of this scoping review for the educational community. Such consultation has been emphasized as a beneficial component in high-quality scoping reviews.^58^ A limitation is the heterogeneity with regard to the CR concept's granularity, different terminology, and lack of formal CR definitions used across health professions. Despite efforts to include relevant studies, the search may have missed articles that referred to the phenomenon of CR without using this terminology. Whereas the study's focus is the overall curricular strategy for CR in an educational program, many articles used “curriculum” in a more delimited sense for CR activities within a limited part of the educational program, focusing on describing content or assessing its effectiveness and not primarily describing the overall CR educational strategy of the program. Our review did not include books, book chapters, dissertations, or non-English articles, which could have revealed different patterns from the ones we found. Our review was guided by an overall aim and 3 specific review questions while recent methodological guidance recommends authors to have one primary review question.^59^ Such a primary question might have provided a more robust structure for the scoping review and resulted in more focused findings.

## Conclusions

This scoping review contributes to increased understanding of CR integration into health professions education curricula. Most curricula address CR in relation to a specific aspect of CR or a limited part of the educational program, meaning that strategies for overarching CR curriculum design throughout an educational program is largely missing in the current CR literature. Information gathering, diagnostics, treatment planning, and students’ self-reflection are frequently described aspects of CR teaching, while aspects of patient participation and interprofessional CR are rare. Educational strategies in CR curricula focus on active and experiential learning approaches, in line with current best evidence. CR assessment methods vary and highlight the need for diverse methods to cover various aspects of CR. These findings offer insights for educators into how CR can be taught and assessed, but they also suggest the need for further guidance on various educational strategies and assessments that should be used as learners progress through an educational program.

## Appendices

### Appendix 1

Search strings used in the database searches

#### PubMed

(Clinical reasoning[Ti] OR Problem solving[Ti] OR reasoning[Ti] OR decision making[Ti] OR judgment[Ti] OR judgement[Ti] OR narrative reasoning[Ti] OR diagnostic[Ti] OR clinical thinking[Ti] OR Management[Ti] OR Cognitive bias[Ti] OR medical error[Ti] OR Clinical competence[Ti] OR Heuristics[Ti]) AND (curriculum[TIAB] OR syllabus[TIAB]) AND (Journal Article[ptyp] AND “last 10 years"[PDat])

#### Web of science

(TI = (Clinical reasoning OR Problem solving OR reasoning OR decision making OR judgment OR judgement OR narrative reasoning OR diagnostic OR clinical thinking OR Patient Management OR Cognitive bias OR medical error OR Clinical competence OR Heuristics) AND TS = (Curriculum OR Syllabus) NOT TS = mathematic*) AND LANGUAGE: (English) AND DOCUMENT TYPES: (Article)

#### CINAHL

TI (Clinical reasoning OR Problem solving OR reasoning OR decision making OR judgment OR judgement OR narrative reasoning OR diagnostic OR clinical thinking OR Management OR Cognitive bias OR medical error OR Clinical competence OR Heuristics) AND AB (curriculum OR syllabus)

### Appendix 2

**Table A2.** Included Studies by Author, Country, Aim, Study Design, Health Profession, and Central Concept(S) Used in the Studies.

Author, year	Country^a^	Aim	Study design^b^	Health profession	Central concept
Charlin et al, 2012^36^	Canada	(i) Develop a new, comprehensive representational model of clinical reasoning processes using Modelling using Typified Objects (MOT) Plus software intended for use by teachers and learners, and (ii) provide data on the validity of this model.	Qualitative	Medicine	Clinical reasoning
Christensen et al, 2017^48^	USA	Explore how clinical reasoning is currently defined, taught, and assessed in the professional education of physical therapists.	Mixed methods	Physical therapy	Clinical reasoning
Dalton et al, 2015^32^	Australia	Describe the development and implementation of an innovative Diploma of Nursing curriculum for preparing Enrolled Nursing students for acute care nursing practice.	Descriptive	Nursing	Clinical reasoning
Daniel et al, 2019^27^	USA	Explore the existing menu of clinical reasoning assessments.	Scoping review	Medicine	Clinical reasoning, Clinical decision-making, Clinical competence
Dowding et al, 2012^19^	UK	Review theoretical concepts that provide a framework for decision making in nursing, as well as methods by which it can be taught.	Narrative review	Nursing	Clinical reasoning, Clinical decision-making, Clinical judgment, Critical thinking
Elvén et al, 2019^49^	Sweden	Explore the associations among the independent variables—knowledge, cognition, metacognition, psychological factors, contextual factors, and curriculum orientation vis-a-vis behavioral medicine competencies—and the dependent variables—outcomes of input from client, functional behavioral analysis, and strategies for behavior change as levels in physical therapist students’ clinical reasoning processes.	Quantitative	Physical therapy	Clinical reasoning
Findyartini et al, 2016^42^	Indonesia, Australia	Explore the influence of the culture of learning on clinical reasoning teaching and learning in two medical schools in Australia and Indonesia, drawing on Hofstede's cultural dimensions.	Mixed methods	Medicine	Clinical reasoning
Furze et al, 2015^20^	USA	Explore the development of student clinical reasoning abilities over time in one professional Doctor of Physical Therapy program.	Qualitative	Physical therapy	Clinical reasoning, Clinical decision-making, Critical thinking
Gillespie, 2010^43^	Canada	The Situated Clinical Decision-Making framework is presented as a tool that provides clinical educators with a structured approach to analyzing nursing students’ and novice nurses’ decision-making in clinical nursing practice, and assists them in identifying specific issues within nurses’ clinical decision-making. The framework also guides educators in selecting relevant strategies to support development of clinical decision-making.	Descriptive	Nursing	Clinical decision-making
Harendza et al, 2017^4^	Germany	Develop and implement a clinical reasoning course in the final year of undergraduate medical training.	Quantitative	Medicine	Clinical reasoning, Critical thinking
Hensel and Billings, 2020^30^	USA	Describe and present strategies to teach and assess the National Council of State Boards of Nursing (NCSBN) clinical judgement model to better help students to achieve entry-level competency.	Descriptive	Nursing	Clinical judgment
Kantar and Alexander, 2012^31^	Lebanon	(i) Explore perceptions of preceptors about the clinical judgment abilities of new baccalaureate nursing graduates. (ii) Understand the influence of curriculum on the development of judgment. (iii) Identify the nature of the gap between practice demands and program outcomes.	Qualitative	Nursing	Clinical judgment
Khin-Htun and Kushairi, 2019^34^	UK	Address practical methods to develop clinical reasoning throughout the whole medical school. Formulate twelve practical strategies to successfully implement teaching of clinical reasoning skills in the pre-clinical and clinical stages.	Descriptive	Medicine	Clinical reasoning
Kononowicz et al, 2020^5^	International	Provide an international perspective on how clinical reasoning is taught and assessed in curricula and to identify the perceived needs and barriers from the teacher's perspective.	Quantitative	Medicine	Clinical reasoning
Kuiper, 2013^21^	USA	Integrate clinical reasoning pedagogies across a baccalaureate curriculum designed to promote a beginner level of competence in solving patient problems.	Descriptive	Nursing	Clinical reasoning
Marcum, 2012^38^	USA	Develop a model of clinical reasoning that integrates both the non-analytic and analytic processes of cognition, along with metacognition	Commentary	Medicine	Clinical reasoning, Clinical decision-making
Menezes et al, 2015^47^	Brazil	Analyze the current state of knowledge on clinical reasoning in nursing undergraduate education.	Scoping review	Nursing	Clinical reasoning, Clinical decision-making, Critical thinking
Nafea and Dennick, 2018^41^	Saudi Arabia, UK	Explore the perceptions of undergraduate dental students regarding clinical reasoning skills and also discover the influences of different curriculum designs on the acquisition of these skills by students.	Qualitative	Dentistry	Clinical reasoning
Orban et al, 2017^35^	Sweden	Monitor and describe students’ progression in professional clinical reasoning skills during health sciences education using observations of group discussions following the case method	Qualitative	Occupational therapy, Speech-language therapy, Midwifery	Clinical reasoning
Pinnock and Welch, 2014^37^	Australia	Consider current theories of clinical reasoning based on research over the last 40 years, its importance in current practice, methods to teach and assess it, common errors in reasoning and suggestions of how to teach it in daily practice.	Narrative review	Medicine	Clinical reasoning
Pinnock et al, 2020^39^	New Zeeland	Introduce medical educators to how artificial intelligence could influence the teaching of clinical reasoning in the undergraduate curriculum.	Commentary	Medicine	Clinical reasoning
Posel et al, 2015^33^	Canada	Describe how previously identified strategies to support the development of clinical reasoning in other settings, can be adapted to, and integrated within, Virtual Patients (VP).	Descriptive	Medicine, Nursing	Clinical reasoning
Rencic et al, 2017^6^	USA	(i) Investigate the content and format of clinical reasoning teaching in US medical schools. (ii) Determine clerkship directors’ attitudes toward clinical reasoning curricula and to identify barriers to the adoption of such curricula.	Quantitative	Medicine	Clinical reasoning
Schaye et al, 2019^29^	USA	Test the theory that a clinical reasoning curriculum grounded in dual process theory and script theory would result in an improvement in the diagnostic reasoning process of residents as assessed by the diagnostic thinking inventory and application of core concepts taught in the curriculum.	Quantitative	Medicine	Clinical reasoning, Diagnostic reasoning process
Schmidt and Mamede, 2015^40^	The Netherlands	Review the variety of approaches employed in the teaching of clinical reasoning and to present a proposal to improve these practices.	Narrative review	Medicine	Clinical reasoning
Stickley, 2018^46^	USA	(i) Describe the design of a planned sequence of courses to teach clinical reasoning to professional physical therapist students using Fink's curriculum design process. (ii) present student outcomes related to the clinical reasoning process.	Descriptive and quantitative	Physical Therapy	Clinical reasoning, Clinical decision-making, Clinical judgment

^a^ Country of the study or country of the first author.

^b^ Study design: Quantitative; Qualitative; Mixed methods; Systematic Review (meta synthesis or meta-analysis); Scoping Review (includes an analytical reinterpretation of the literature); Narrative Review (no analytical reinterpretation of the literature); Descriptive (a description of for example a course, methods or practical tips); Commentary.
